# Advances in Identifying Beryllium Sensitization and Disease

**DOI:** 10.3390/ijerph7010115

**Published:** 2010-01-13

**Authors:** Dan Middleton, Peter Kowalski

**Affiliations:** 1 Division of Health Studies, Agency for Toxic Substances and Disease Registry, CDC Chamblee Campus, Building 106, 4770 Buford Highway NE Mail Stop F-57, Atlanta, GA 30341, USA; 2 Division of Health Assessment and Consultation, Agency for Toxic Substances and Disease Registry, CDC Chamblee Campus, Building 106, 4770 Buford Highway NE Mail Stop F-59, Atlanta, GA 30341, USA; E-Mail: pek2@cdc.gov

**Keywords:** beryllium, BeLPT, beryllium sensitization, BeS, screening, chronic beryllium disease, CBD

## Abstract

Beryllium is a lightweight metal with unique qualities related to stiffness, corrosion resistance, and conductivity. While there are many useful applications, researchers in the 1930s and l940s linked beryllium exposure to a progressive occupational lung disease. Acute beryllium disease is a pulmonary irritant response to high exposure levels, whereas chronic beryllium disease (CBD) typically results from a hypersensitivity response to lower exposure levels. A blood test, the beryllium lymphocyte proliferation test (BeLPT), was an important advance in identifying individuals who are sensitized to beryllium (BeS) and thus at risk for developing CBD. While there is no true “gold standard” for BeS, basic epidemiologic concepts have been used to advance our understanding of the different screening algorithms.

## 1. Background

Beryllium (Be) is a lightweight metal with valuable physical and chemical properties that include stiffness, corrosion resistance, and electrical and thermal conductivity [1]. Since the first commercial use in the 1920s, it has been used in a growing number of commercial and defense applications, including aircraft and satellite structures, nuclear applications, precision instruments, and high speed electronic circuits [2].

### 1.1. Early Disease Reports

European researchers first linked beryllium exposure to occupational lung disease in the 1930s [3]. Beginning in the 1940s, researchers in the United States showed that chronic beryllium disease (CBD) was an immunologically mediated granulomatous lung disease that could result from the inhalation of airborne beryllium particles [2].

Although a recent paper argues otherwise [4], most researchers have distinguished between acute and chronic beryllium disease. Acute beryllium disease results from an irritant response at high exposure levels, whereas chronic beryllium disease (CBD) typically results from a hypersensitivity response to lower exposure levels. This article provides an overview of advances in identifying beryllium sensitization and disease in the United States.

### 1.2. The Beryllium Registry

To clarify the epidemiology and natural history of beryllium disease, the U.S. Beryllium Case Registry was established in 1952 at the Massachusetts Institute of Technology. It later moved to the National Institute for Occupational Safety and Health (NIOSH) and is now closed. Adding a case to the Registry required documentation of beryllium exposure plus any three of the four criteria summarized below [5,6]:

1. lower respiratory symptoms;
2. reticulonodular infiltrates on chest x-ray;
3. restrictive or obstructive pulmonary impairment, or depressed diffusing capacity;
4. biopsy showing non-caseating granulomas or mononuclear cell interstitial infiltrates.

Approximately 900 cases were entered into the registry, including 65 cases among family members exposed to “worker take home” dust and residents exposed through off-site air pollution. Unfortunately, the registry criteria were not sufficiently specific to fully distinguish between chronic beryllium disease and sarcoidosis, a clinically similar granulomatous lung disease that is not associated with beryllium [7].

### 1.3. Exposure and Susceptibility

To develop CBD, an individual must be exposed and then develop immunologic sensitization to beryllium (BeS) over a period of months or years. The physical form and physicochemical properties may play a role in beryllium toxicity [8,9]. In addition to inspired beryllium particles, researchers have suggested that skin contact with fine beryllium dust may play a role in sensitization [10].

In 2004, NIOSH estimated that up to 134,000 workers in the United States were exposed to beryllium [11]. The Occupational Safety and Health Administration (OSHA) Permissible Exposure Limit for beryllium in air is 2.0 micrograms per cubic meter averaged over an eight-hour work day. This exposure limit is widely recognized as failing to protect exposed workers from sensitization and chronic beryllium disease [12]. In addition, there are currently no requirements that prevent general industry beryllium workers from inadvertently taking beryllium dust home on their clothing.

Recent cross-sectional studies indicate that the prevalence rates of BeS and CBD among beryllium workers lie between 5−21% and 3−21%, respectively [12]. A growing body of evidence indicates that genetic susceptibility of the exposed workers also contributes to beryllium disease outcomes [13].

### 1.4. The BeLPT

CBD case identification was improved by the development and spread of the beryllium lymphocyte proliferation test (BeLPT) in the early 1980s. The BeLPT can detect evidence of beryllium sensitization in the blood before there is evidence of pulmonary disease. Abnormal BeLPT results identify individuals at higher risk for disease and typically lead to further medical evaluation to rule out CBD.

To perform the BeLPT, T-lymphocytes are incubated in three concentrations of beryllium sulfate over two time periods for a total of six “exposed” incubations. The beryllium sulfate stimulates sensitized T-lymphocytes to take up tritiated thymidine. The radiation levels are compared for incubations with and without beryllium sulfate. The relative radiation levels for the BeLPT are interpreted as follows:

- normal test result (NL)—0 of 6 incubations with Be are elevated;
- borderline test result (BL)—1 of 6 incubations with Be are elevated; and,
- abnormal test result (AB)—2 or more incubations with Be are elevated.

### 1.5. Test-Retest Inconsistencies

Deubner *et al*. [14] explored issues associated with the validity of the BeLPT in an article published in 2001. They noted substantial intra- and inter-laboratory disagreement among the laboratories evaluated. Concern about these inconsistencies has led practitioners to require serial testing and various combinations of test results to confirm sensitization to beryllium.

## 2. Introduction

### 2.1. The Expert Panel

On April 26, 2006, ATSDR convened an expert panel in Ottawa County, Ohio [15]. The seven member panel included physicians with beryllium expertise who were associated with environmental activism, industry, and universities or government. The panel was asked to provide input to ATSDR on various issues related to the BeLPT. When asked about the criteria that provided sufficient BeLPT evidence of sensitization for referral and medical evaluation, the various panelists’ suggested criteria that included:

- one abnormal result,
- one abnormal and one borderline result, and,
- two abnormal results.

Two abnormal results have historically been used in lieu of a “gold standard” for beryllium sensitization in beryllium research [14,16], though it is not as sensitive as other criteria. Using probability modeling, a previous manuscript considered the overall sensitivity, specificity, and positive predictive values for true BeS for the three different criteria (above) in common use [17].

The exposure context is also important when interpreting results for the individual. That is, the exposure level alters the expected group prevalence and therefore affects the positive predictive value of individual test results.

### 2.2. Confusing Issues

One thing evident from the panel’s deliberations was a lack of consensus on the “best” criteria for BeS, or even on what testing characteristics were the most important. For example, *one abnormal* is the most sensitive criterion, but *two abnormals* provide more specificity. The overall sensitivity, specificity, and predictive values of the various criteria and associated algorithms for BeS were largely unknown. In this review, we use these terms generally to mean:

*Sensitivity*—the proportion of persons with BeS that test abnormal;
*Specificity*—the proportion of persons without BeS who test normal;
*Positive predictive value (PPV)* – the proportion of persons testing abnormal who are truly BeS.

Estimates of these parameters are needed to support informed decisions regarding referral for medical evaluation. Further, the following question remained to be addressed: *Is there a background level of BeS in the general population?*

## 3. Probability Modeling

As noted, there is no true gold standard for BeS. Given that the BeLPT is only moderately sensitive but highly specific for BeS, Stange *et al*. [16] found an ingenious way to circumvent this problem. It follows from these epidemiologic characteristics that *usually*:

- if a true positive occurs, additional testing will confirm it; and,
- if a false positive occurs, additional testing will not confirm it.

This allowed Stange to propose a working gold standard based on serial testing to confirm (or not) any abnormal result. That is, if a total of two abnormals were documented, the individual was identified as BeS; if not, the first abnormal was considered a false positive. This provided the data necessary to evaluate the sensitivity and specificity of the single BeLPT test, the building blocks for modeling the performance of testing algorithms and associated criteria for BeS.

### 3.1. Building a Foundation

Stange *et al*. [16] reported that a single BeLPT’s sensitivity was 0.683 and the specificity was 0.969. Middleton *et al*. [18] adjusted Stange’s parameters to consider borderline test results and found that, for persons *truly sensitized* to beryllium, the single test outcome probabilities are: P_AB_ = 0.5970; P_NL_ = 0.2770; and, P_BL_ = 0.1260. Similar calculations can be done for persons *not truly sensitized* to beryllium; in this group, the single test outcome probabilities are: P_AB_ = 0.0109; P_NL_ = 0.9733; and P_BL_ = 0.0158.

### 3.2. Probability Modeling

**Figure 1.**
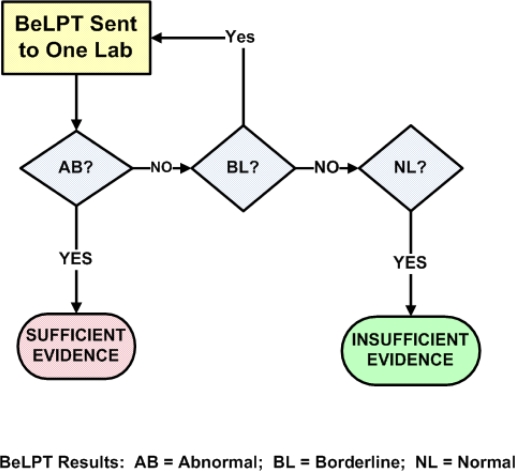
One abnormal BeLPT provides sufficient evidence for beryllium sensitization.

Since we know that the probability of one coin toss resulting in one head (or one tail) is 1/2, we calculate the probability of getting a head and then a tail as follows: p = 1/2 * 1/2 = ¼; that is, p = 0.25. The same logic can quantify the modeling specified in various testing algorithms. For example, consider Figure 1 and Table 1.

Note that “Combination 1” is nothing more than an abnormal result on “Test 1”, which requires no additional testing. The probability of this outcome is simply the single test probability that a truly sensitized person will have an abnormal result on any particular test (0.5970). Using the algorithm in Figure 1, note that borderline test results are simply repeated. Combination 2 requires a borderline (p_BL_ = 0.1260) followed by an abnormal (p_AB_ = 0.5970); the probability of this combination is the product of these probabilities, or p = 0.1260 * 0.5970 = 0.0752. Combination 3 requires a borderline, then a second borderline, and finally an abnormal; the product of these three probabilities is 0.0095. Adding these three probabilities yields 0.6817, the probability of meeting the criteria if the individual is truly BeS. This is also the algorithm’s overall “sensitivity”.

**Figure 2.**
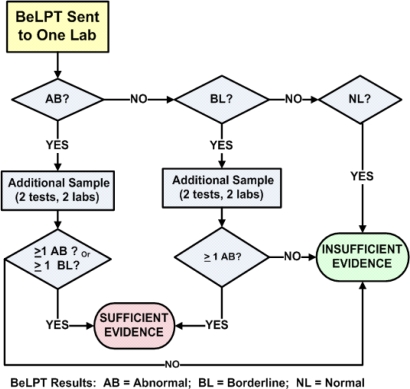
One abnormal and one borderline provide sufficient evidence for beryllium sensitization.

**Figures 3.**
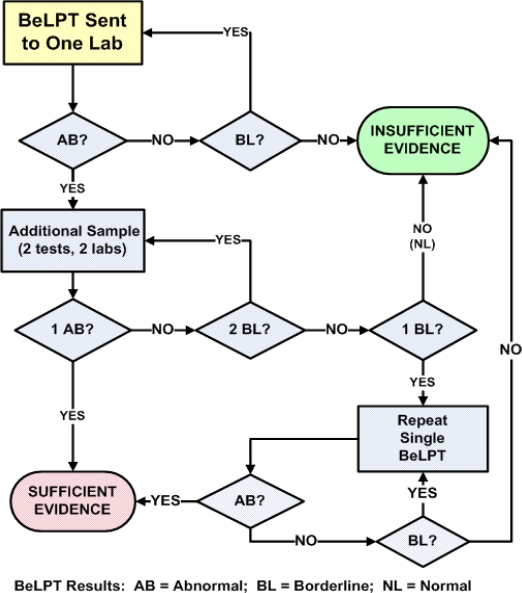
Two abnormal BeLPTs provide sufficient evidence for beryllium sensitization.

The individual tables for Figures 2 and 3 are not presented in this review, but are published elsewhere [17]. The overall characteristics for each algorithm/criteria are shown in Table 2.

### 3.3. The National Research Council (NRC)

Government agencies have differed greatly in their approach to protecting workers from the health effects associated with exposure to beryllium. The U.S. Department of Energy has a comprehensive program for hygiene, housekeeping, personal protective equipment, and testing for current and former workers. Historically, Department of Defense policy statements have discouraged use of the BeLPT for the screening or surveillance of exposed workers [15]. However, more recently the U.S. Air Force asked a committee of the National Research Council (NRC) to conduct an independent review of the scientific literature on beryllium and to make recommendations for exposure- and disease-management [18]. Committee members had expertise in pulmonary medicine, occupational medicine, epidemiology, industrial hygiene, toxicology, pathology, biostatistics, and risk assessment.

Among the topics covered in the 2008 NRC report was the role of the BeLPT in worker surveillance. The committee concluded that:

“The BeLPT is integral to any screening program. No alternative tests have been adequately validated to be put into practice outside research settings.”

The algorithm recommended to the Air Force was taken from the article by Middleton *et al*. [15]. This algorithm is based on the criteria of “one abnormal plus one borderline.” Citing Stange *et al*. [16] and Middleton *et al*. [15,17], the NRC committee also advised consideration of the prevalence of BeS in the population being tested.

Finally, the NRC noted that current practice is not always strictly limited to a specific algorithm, but considers results collected at various times over long time periods. For example, one *abnormal* BeLPT result that is followed first by two normals and then later by another *abnormal,* is considered equivalent to two sequential *abnormals* and the individual is referred for evaluation. The committee acknowledged some uncertainty, but suggested that the current approach continue until data is available to clarify the impact of such practices.

## 4. Validation

The question of interest is: *“How well does it predict what will happen when a different group of individuals is tested?”.* To date, the sensitivity and positive predictive value estimates are based on the exposed workers studied by Stange *et al*. [16] and have not been compared to the screening experience in other populations. However, some simple checks on the *specificity* of the BeLPT are readily available and are described below.

### 4.1. DOE Pre-hires at Rocky Flats

In addition to the exposed workers, Stange *et al*. [16] also studied 291 new hires who had not yet been occupationally exposed to beryllium. While three of the 291 (1%) had an abnormal BeLPT result, none of the three individuals with abnormal results was confirmed by a second abnormal. While this doesn’t tell us anything about the sensitivity of the BeLPT, it does suggest that confirmed results provide specific evidence for beryllium sensitization. That is, false positives do not seem to be a big problem when confirmation is required by the criteria.

### 4.2. Manatee County, FL

ATSDR tested 114 residents of Manatee County who lived near a machine shop that used beryllium; none were workers or household contacts of workers. There were one abnormal (0.88%) and two borderline (1.75%) results during the first round of testing. Given that the machine shop had no stack emissions, this population was probably unexposed and unsensitized. For such a population, the estimates developed in Middleton *et al*. [17] predict 1.09% abnormal and 1.58% borderline results. As also predicted by the model, none of these abnormal results were confirmed as BeS during follow-up testing.

### 4.3. Japan

Yoshida *et al*. tested 159 new hires in Japan. Of these unexposed workers, two (1.26%) had an abnormal result, consistent with a predicted rate of 1.09%. No information about follow-up testing to confirm sensitization was provided. Insofar as we can tell, these results are consistent with those among unexposed persons in the United States.

### 4.4. Ottawa County, OH

While the remaining beryllium producer in the United States has reported a low prevalence of beryllium sensitization among new hires (~1%) [19], the consistent lack of confirmed sensitization among other groups seems more likely to be representative of the general population. Through local hiring and hiring relatives of current workers, some of the new hires at this facility may have had prior exposure to beryllium. Further, confirmation testing at this facility is generally conducted after beginning work at the plant.

## 5. Conclusions

### 5.1. Modeling vs. Measurement

As suggested by the NRC committee, testing does not always follow a strictly defined protocol. This clearly has implications for predictions generated by the models, but the specifics are not well understood. One useful approach would be to measure the actual predictive value of various combinations of results in a specific exposed population for identifying persons eventually determined to be sensitized to beryllium. The relationship between various combinations of BeLPT results and eventually developing CBD, if one exists, also has yet to be clarified. That is, within the same algorithm, are specific combinations confirming sensitization more predictive of CBD than others?

### 5.2. Not Truly Sensitized

The test results for persons not exposed (and therefore *not truly sensitized*) appear to be consistent with the models’ predictions. Screening BeLPT results among unexposed persons are rarely abnormal (1.09%), rarely borderline (1.58%), and in most populations, not confirmed by followup testing when they do occur. That is, the sensitization criteria that require confirmation seem to be very specific for BeS.

### 5.3. Truly Sensitized

Similar evaluations need to be done with persons who are truly sensitized to validate that the algorithms function as predicted in identifying persons truly sensitized to beryllium. Further, grouping outcomes by those that meet the minimum criteria results (e.g., 1 AB + 1 BL + 1 NL) is not entirely satisfactory, since the positive predictive values calculated apply to the algorithm outcomes as a whole rather than to any specific outcome. Future publications by the authors and other collaborators will attempt to clarify these issues.

## Figures and Tables

**Table 1.** Likelihood of Meeting BeS Criteria (One Abnormal BeLPT) for Individuals Truly Sensitized^1^.

**Results that Meet Criteria^2^**	**Test 1**	**Test 2**	**Test 3**	**Outcome Probability** p = p_1_ * p_2_ * p_3_
**Combination 1**	*abnormal*	---	---	0.5970
**Combination 2**	*borderline*	*abnormal*	---	0.0752
**Combination 3**	*borderline*	*borderline*	*abnormal*	0.0095
Overall likelihood of meeting the criteria of one abnormal				0.6817

1 The single test probabilities for persons truly sensitized [17] are p_AB_ = 0.5970, p_BL_= 0.1260, and p_NL_= 0.2770.

2 Test Results that Meet Criteria” are groups of 1, 2, or 3 test results that combine to meet the sensitization criteria specified (in this case, *one abnormal BeLPT*).

**Table 2.** Overall Sensitivity, Specificity, and Positive Predictive Value, by the Specified Sensitization Criteria^1^.

**Sensitization Criteria**	**Sensitivity**	**Specificity**	**PPV at4% BeS 2**
**1 AB**	0.682	0.9889	0.719
**1 AB + 1 BL**	0.657	0.9992	0.972
**2 AB**	0.612	0.9998	0.992

1 Positive predictive value (PPV) also varies by prevalence; the PPV’s shown are for a population with 4% prevalence of BeS.

2 The specified sensitization criteria imply the groups of results combinations that are evaluated together. The nominal criteria is actually the minimum.
